# Reverse Vaccinology Approach in Constructing a Multi-Epitope Vaccine Against Cancer-Testis Antigens Expressed in Non-Small Cell Lung Cancer

**DOI:** 10.31557/APJCP.2021.22.5.1495

**Published:** 2021-05

**Authors:** Leana Rich M. Herrera

**Affiliations:** *Department of Physical Sciences, College of Science, Polytechnic University of the Philippines, Manila City, Philippines. *

**Keywords:** NSCLC, reverse vaccinology, in silico, cancer-testis antigen

## Abstract

**Background:**

The 5-year survival rate of non-small cell lung cancer (NSCLC) patients has not significantly improved despite advancements in the currently applied treatments. Thus, efforts are put forth in developing novel immunotherapeutic agents targeting cancer-testis antigens (CTA) in NSCLC. This work utilized reverse vaccinology approach in designing a novel multi-epitope vaccine targeting melanoma-associated antigen 3 (MAGEA3), MAGEA4, New York esophageal squamous cell carcinoma-1 (NY-ESO-1), and Kita-Kyushu lung cancer antigen 1 (KK-LC1), being the most frequently expressed CTAs in NSCLC.

**Methods:**

Epitopes were mapped from the sequences of CTAs. The population coverage (PC) of identified CD4+ and CD8+ epitopes were estimated. Candidate linear B cell (BL), CD4+, and CD8+ epitopes were adjoined in a multi-epitope construct (Mvax) with flagellin domain as an adjuvant. Antigenicity, and cross-reactivity of Mvax were examined. The tertiary structure of Mvax was modelled, and validated. All epitopes included in the vaccine were docked with their human leukocyte antigen (HLA) binders. The immunogenicity of epitopes in Mvax was validated through molecular dynamics analysis.

**Results:**

Mvax contains 22 epitopes from MAGEA3, MAGEA4, NY-ESO-1, and KK-LC1. It is classified as antigenic, non-allergen, non-toxic, and possesses physicochemical stability. Epitopes have no significant hits with other human proteins, except for 2 other CTAs frequently expressed in NSCLC. The stretch of BL epitopes in Mvax confers flexibility, and accessibility emphasizing its antigenicity. The tertiary structure analysis showed that Mvax model has good structural quality. All epitopes included in the vaccine are highly immunogenic as indicated by favorable binding affinity, low binding energy, and acceptable root-mean-square deviation (RMSD). CD4+ and CD8+ epitopes have global PC of 81.81%, and 84.15%, respectively.

**Conclusion:**

Overall, in silico evaluations show that Mvax is a potential immunotherapeutic agent against NSCLC.

## Introduction

Lung cancer is the primary cause of cancer-related deaths in the world with non-small cell lung cancer (NSCLC) making up 84% to 87% of the lung cancer cases (Bray et al., 2018). Current treatments include radiation therapy, surgery, chemotherapy, monoclonal antibodies, or the combination of such. However, these methods are often accompanied by adverse reactions, metastasis, and tumor regression. Despite the advancements made in the last decades, the 5-year survival rate of lung cancer patients remains poor (Smida et al., 2020). 

One of the alternative treatments used for lung cancer is immunotherapy. This approach takes advantage of the patient’s immune system by training it to recognize, and respond to antigens expressed by cancer cells, resulting to tumor-mechanism inhibition or direct cytotoxicity to cancer cells. To develop effective immunotherapeutic agents, cancer antigens should be identified and strategically targeted. Tumor antigens can be overexpressed proteins, mutated gene products, or preferentially expressed in cancer cells other than normal cells such as the cancer-testis antigens (CTA) (Jin et al., 2018). The expression of CTAs in normal tissues is restricted to the testis, ovaries (Salmaninejad et al., 2016), and are widely expressed in several human cancer tissues. CTAs have been shown to induce humoral or cellular antitumor responses in cancer patients (Rosenzweig et al., 2012; Raza et al., 2020). Nevertheless, autoimmunity is prevented due to the downregulation of human leukocyte antigen (HLA), and the expression of immunosuppressors (Janitz et al., 1994; Hunt, 2006) in placenta, ovary, and testis. Well-studied CTAs include the members of melanoma-associated antigen (MAGE) family. The expression of *MAGEA* genes has been strongly associated with lung cancer (Bhan et al., 2011). MAGEA3 is one of the most frequently expressed CTAs in NSCLC (Hanagiri et al., 2013; Keshavarz-Fathi and Rezaei, 2020). Similar to MAGEA3, MAGEA4 is also frequently expressed in NSCLC patients (Chen et al., 2013; Hou et al., 2020). Another commonly expressed CTA is the New York esophageal squamous cell carcinoma-1 (NY-ESO-1), also known as cancer/testis antigen 1. It is encoded by gene cancer/testis antigen A1 (*CTAGA1*) which is expressed in testis, ovary, and various cancer tissues such as NSCLC. NY-ESO-1 is currently known as the most immunogenic CTA (Gjerstorff et al., 2013; Xia et al., 2018; Smith and Iwonofu, 2018). Kita-Kyushu lung cancer antigen 1 (KK-LC1), encoded by cancer/testis antigen 83 (*CT83*), is a single-pass membrane protein normally expressed in testis, and other cancer types including lung cancer (Fukuyama et al., 2006; Jin et al., 2018; Marcinkowski et al., 2019; Ichiki et al., 2020). Strong immunogenicity, tumor-restricted, and biased expression of MAGEA3, MAGEA4, NY-ESO-1, and KK-LC1 can offer vast drug-development opportunities, including immunotherapeutic agents and vaccines. Therefore, the primary goal of this work is to apply reverse vaccinology to efficiently design a novel multi-epitope vaccine that can potentially induce immune response against multiple CTAs expressed in NSCLC. Multi-epitope vaccine can be more advantageous than whole recombinant proteins, or even single peptides. It may cover wider population coverage, and exclude cross-reactive sequences to prevent adverse reactions. With the aid of immunoinformatics, the cost, effort, and time required to develop vaccines, can be minimized (Oyarzún et al., 2016).

## Materials and Methods

*Identification of linear B cell and T cell epitopes*

The amino acid sequences of Homo sapiens MAGEA3 (P43357), MAGEA4 (P43358), NY-ESO-1 (P78358), and KK-LC1 (Q5H943) were retrieved from UniProt Database. Extracellular sequence of KK-LC1 (22-113) was mapped to identify linear B cell (BL) epitopes. ABCPred, and Emini Surface Accessibility (ESA), BepiPred Linear Epitope (BLE), and Kolaskar and Tongaonkar Antigenicity (KTA) tools in the Immune Epitope Database (IEDB) were utilized. Epitopes from ABCPred with overlapping sequences from at least 2 tools in IEDB, were chosen as final BL epitopes. Among the four tumor antigens, only KK-LC1 has an extracellular sequence; thus, it was the only antigen used to identify BL epitopes. The full-length amino acid sequences of MAGEA3, MAGEA4, NY-ESO-1, and KK-LC1 were mapped for T cell epitopes using default thresholds. CD4+ epitopes, and their corresponding major histocompatibility complex (MHC) II binders were identified using NN-align-2.3 (netMHCII-2.3), and NetMHCPanII4.0E in IEDB. A reference list for the most common MHC II binders was utilized (Greenbaum et al., 2011). Consensus CD4+ epitopes (15 residues) with good binding affinity to at least 5 MHC II (IC_50_ ≤ 150nM) in NN-align-2.3, and with rank ≤ 10 in NetMHCIIPan4.0E, were selected as final CD4+ epitopes. CD8+ epitopes and their MHC I binders were identified using NetMHCcons method in the Proteasomal cleavage/TAP transport/MHC class I combined predictor which combines three MHC-peptide binding prediction methods to give more reliable result (Karosiene et al., 2012). A list containing the most frequent MHC I binders was uploaded (Weiskopf et al., 2012). Epitopes with 8-11 residues, binding to at least 5 MHC I, with TAP and proteasome scores > 1.0, and with IC_50_ ≤ 150nM, were selected as final CD8+ epitopes. Peptides with IC_50_<500nM are classified as good binders (Jensen et al., 2018). A multi-epitope vaccine can offer larger population coverage (PC). To estimate global PC, the set of final CD4+ and CD8+ epitopes were queried separately, using the Population Coverage tool in IEDB. 

*Multi-epitope construct*

KK-LC-1 CD4+ epitopes with > 2 residues overlap were shortlisted using BL epitopes as templates. And CD8+ epitopes which overlap with CD4+ epitopes in the same antigen, were shortlisted using CD4+ epitopes as templates. Overlapping epitopes of the same antigen were merged as continuous peptides. BL and CD4+ epitopes were adjoined using GPGPG linkers, and AAY was used to connect CD8+ epitopes. Peptides from the same antigen were arranged next to each other according to their sequence position. Salmonella typhimurium flagellin (fliC) sequence was retrieved from Uniprot database (P06179), and used as an adjuvant. Its N-terminal domain 1 (ND1) region (46-175) was linked to the cluster of CD8+ and CD4+ epitopes, via flexible EAAAK linker. Then, the C-terminus of the construct was fused with the C-terminal D1 (CD1) region of fliC (398-455) using EAAAK. The series of BL epitopes were connected to CD1 of fliC. Lastly, a valine residue was added at the N-terminus of the construct (Mvax) with the aim of increasing its half-life (Herrera, 2020).

*Evaluation of antigenicity, allergenicity, toxicity, cross-reactivity, and physicochemical properties of Mvax*

The whole sequence of Mvax construct was assessed for antigencity in VaxiJen2.0 using ≥0.5 threshold in tumor model. It utilizes an alignment-independent method predicting an antigen with 70%-89% accuracy (Doytchinova and Flower, 2007). The sequence was further evaluated in ANTIGENpro, with estimated accuracy of 76% using cross-validated experiments (Magnan et al., 2010). AllergenFPv1.0 was used to identify potential allergen sequence in Mvax. This tool generates the highest Tanimoto score to the nearest allergenic sequence in the database (Dimitrov et al., 2014). Sequences with exact match to human proteome, other than the target CTAs, may result to cross-reactivity; thus, the sequence of Mvax was queried against human proteome databases using default settings in the protein basic local alignment search tool proteins (BLASTp). The physicochemical properties of Mvax were estimated in sillico in ExPASy ProtParam tool (https://web.expasy.org/protparam/). 

*Secondary structure analysis, and tertiary structure modelling with validation*

The series of B cell epitopes in Mvax must be flexible, and exposed enough for B cell receptors (BCR) to effectively bind to it. In this work, Mvax sequence was evaluated for its secondary structure composition, disordered, accessible, and hydrophilic regions. The position of B cell epitopes were particularly investigated. GOR4 web tool (https://npsa-prabi.ibcp.fr/cgi-bin/npsa_automat.pl?page=npsa_gor4.html) was utilized to evaluate the secondary structure composition of Mvax. Disordered, and solvent-accessible regions were identified in RaptorX (http://raptorx.uchicago.edu/StructurePropertyPred/predict/). GalaxyTBM tool (http://galaxy.seoklab.org/cgi-bin/submit.cgi?type=TBM) was used in generating tertiary structure model for Mvax. GalaxyRefine server (http://galaxy.seoklab.org/cgi-bin/submit.cgi?type=REFINE) was employed to improve resulting tertiary structure model. Different validation tools were employed to check the quality of refined structures. PROCHECK generated Ramachandran plot to show percentage of residues lying within the favoured, and disallowed regions. ProSa-web calculates the z-score of a structure by estimating its deviation from validated x-ray crystallography and NMR structures of native proteins (Wiederstein and Sippl, 2007). Qualitative model energy analysis (QMEAN) score provides an estimate of the degree of nativeness in the structural model by comparing it to experimental structures of similar size. Scores ≤-4.0 indicate a low-quality model (Benkert et al., 2011). Finally, the best structure model for Mvax was chosen and viewed in Pymol. 

*Structural B cell epitopes*

The BL epitopes incorporated in Mvax must be protruded enough so BCRs can bind to it. This work utilized Ellipro to identify structural epitopes on the tertiary structure model of Mvax. As the best structure-based algorithm amongst the others, Ellipro predicts the conformational and the linear epitopes based from protrusion index (PI) of a residue, and provides PI score for each protruded sequence (Ponomorenko et al., 2008). 

*Molecular docking and simulation*

All CD8+ epitopes were docked with their most common HLA I binder(HLA-B*15:01), except for GSVVGNWQYFF which was docked with HLA-B*35:01 having the lowest binding affinity (IC_50 _= 143.49). Most CD4+ epitopes were docked with their most common HLA-DRB1*01:01, while epitopes VLHHMVKISGGPHIS, ERVIKNYKRCFPVIF, and ALIVFWKYRRFQRNT were docked with HLA-DRB1*11:01 as their common binder. PDB structures of HLA-B*15:01 (PDB ID: 6uzs), HLA-B*35:01 (PDB ID: 4lnr), HLA-DRB1*01:01 (PDB ID: 1aqd), and HLA-DRB1*11:01 (PDB ID: 6cpl) were retrieved from the Research Collaboratory for Structural Bioinformatics Protein Data Bank (RCSB PDB). Epitopes were docked to their respective binders using GalaxyPepDock which performance has been proven superior versus other web servers (Lee et al., 2015). Then, GalaxyRefineComplex tool was used to refine the structures of docked complexes, and viewed in iCn3D server (https://www.ncbi.nlm.nih.gov/Structure/icn3d/full.html). The binding energies, and dissociation constants of docked complexes at 37^o^C were calculated in PRODIGY web server which makes use of both intermolecular interactions, and non-interface surface properties (Xue et al., 2016). Molecular dynamics simulation was performed to evaluate the stability of interaction within the complex using plot of root-mean-square deviation (RMSD) per residue. For this process, C-alpha Brownian dynamics was set in 100 ps time, time change 0.01 ps, 3.8Ǻ distance between alpha carbon atoms, output frequency of 10 steps, and force constant of 40 kcal/mol Ǻ2. It was performed in MDWeb server (http://mmb.irbbarcelona.org/MDWeb/) which employs force-field Amber-99sb in GROMACS MD setup with solvation (Hospital et al., 2012).

## Results

*B cell and T cell epitopes*

BL epitopes with residues 1-20 were mapped from the extracellular sequence of KK-LC1 (22-113). BL epitopes (16 residues) from ABCPred tool which overlapped with sequences from at least 2 tools in IEDB are KK-LC1 sequences 22-37, 77-92, and 89-113. Finally, sequences were merged to 22-37, and 77-113, and were included in the vaccine construct. Table 1 shows 12 final CD4+ epitopes identified in MAGEA3 (3), MAGEA4 (3), NY-ESO-1 (3), and KK-LC1 (3). The estimated global population for the set of CD4+ epitopes is 81.81%. Seven candidate CD8+ epitopes (Table 1) and their corresponding MHC II binders were mapped from MAGEA3 (4), MAGEA4 (2), and NY-ESO-1 (1). KK-LC1 CD8+ epitopes were not included because the predicted epitopes did not meet the criteria used in this work. Estimated worldwide population using the set of CD8+ epitopes is 84.15%.

*Mvax construct*

**Figure 1:**
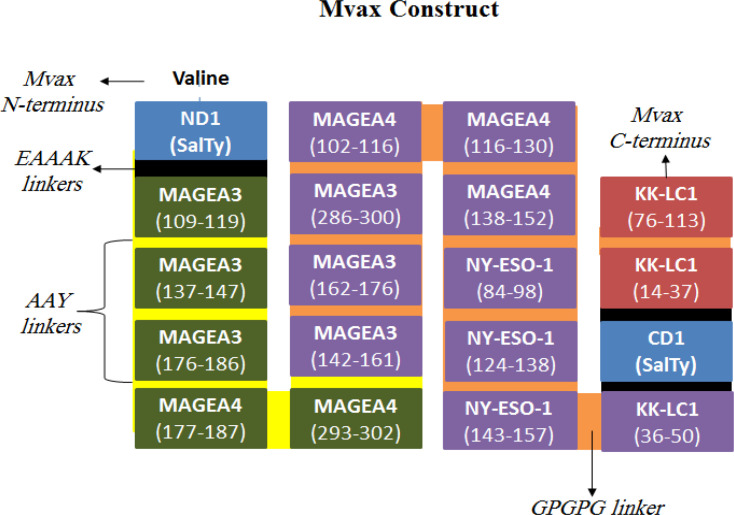
Diagrammatic Presentation of Mvax Construct

The schematic presentation of the whole Mvax construct (Figure 1) shows the valine residue at the N-terminus of fliC ND1 (blue). Epitopes were arranged in the multi-epitope construct starting from CD8+ (green), CD4+ (purple), fliC CD1 (blue), and BL epitopes (maroon). Some KK-LC1 B cell epitopes can also function as CD4+ epitopes (14-28 and 76-90) due to sequence overlap. Other CD4+ sequences can also function as CD8+ epitopes, including NY-ESO-1 CD4+ sequence (84-98) for CD8+ epitope (89-96), and MAGEA3 CD4+ sequrnce (147-161) for CD8+ (142-151). 

*Antigenicity, allergenicity, toxicity, cross-reactivity, and physicochemical properties of Mvax*

Mvax is validated antigenic in Vaxijen server (0.5956), and in ANTIGENpro (0.903634). It is classified as non-allergen, having the highest Tanimoto similarity index of 0.86 with Q96PE2. Potentially toxic sequences were not mapped in the vaccine. Results showed that four epitopes have exact sequence match with other known CTAs—MAGEA6 and CTAG2. These include MAGEA3 CD8+ 137-147, 142-151, 176-186 with MAGEA6; and NY-ESO-1 CD4+ 143-157 with CTAG2. More importantly, none of the epitopes has significant match with other human proteins in the databases. Mvax construct has 540 residues with molecular weight of 57,839.58 Da, and 9.23 theoretical pI. The estimated half-life is 100 hours (mammalian reticulocytes, in vitro), >20 hours (yeast, in vivo), and >10 hours (*E. coli*, in vivo). Its instability index is 31.55, aliphatic index (thermostability) is 78.08, and its grand average hydropathicity (GRAVY) is -0.156 which classifies it as slightly hydrophilic. 

*Secondary structure composition and tertiary structure model of Mvax*

**Figure 2:**
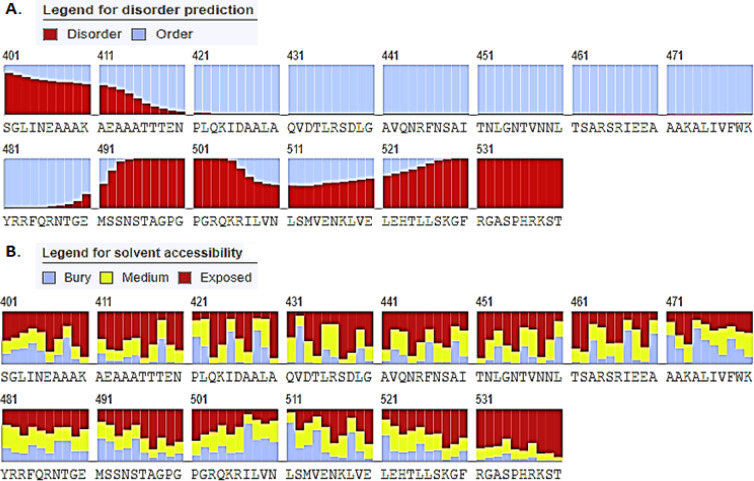
Disordered and Solvent-Accessible Residues in Mvax

**Figure 3:**
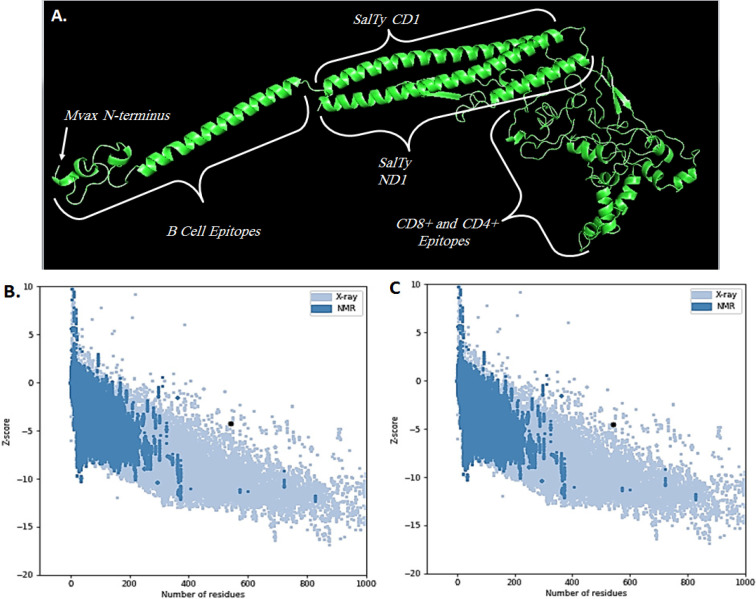
Mvax Tertiary Structure Model (A) viewed in Pymol, and z-score plots for unrefined (B) and refined structures (C)

Mvax is consist of 16.67% extended strands, 39.63% alpha helix, and 43.70% random coil. Figure 2 shows that the residues of BL epitopes (474-540) lie within the disordered region of Mvax (2A), and have medium to full exposure (2B). The tertiary structure model of Mvax has improved after refinement (Figure 3A). The residues within the favoured regions increased (90.1% to 91.7%), and decreased in the disallowed regions (1.5% to 1.3%). The QMEAN score improved from -4.22 to -3.38, indicating a better quality for the refined model. The z-score of Mvax from -4.22 (Fig.3B) to -4.52 (Figure 3C), moved closer to z-scores of native proteins.

*Structural epitopes of Mvax*

Table 2 shows that the series of BL epitopes (474-540) in the tertiary structure of Mvax, can both function as linear and discontinuous structural epitopes. These sequences are extremely protruded, as indicated by high protrusion scores.

*Docking and molecular dynamics simulation*

**Figure 4:**
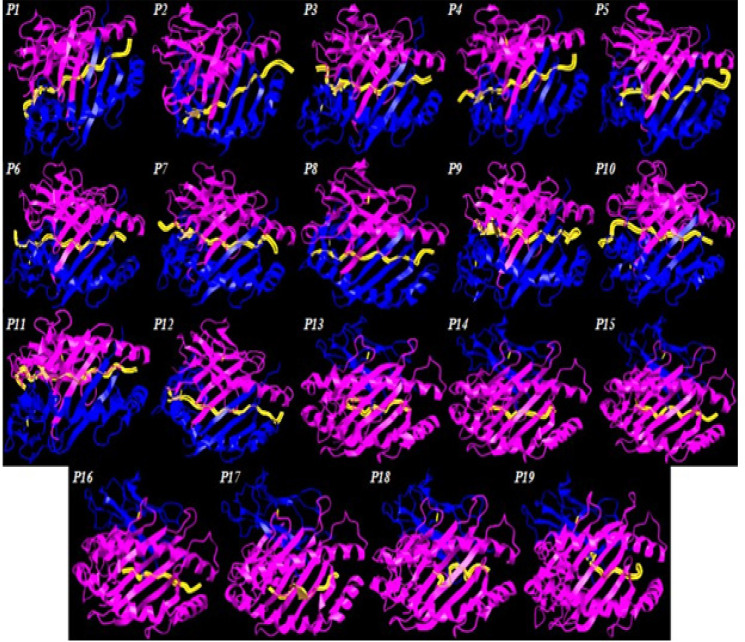
Epitope-HLA Docked Structures Viewed in iCn3D. CD4+ (P1-P12) and CD8+ (P13-P19) Epitopes (Yellow) are Docked within the Binding Pockets of HLA Heterodimer Structures (in Purple and Blue)

Figure 4 shows all CD4+ and CD8+ epitopes docked within the binding groove of HLA. The formation of epitope-MHC docked complexes are energetically favoured (Table 3), wherein the highest is P15 (GSVVGNWQYFF-HLA B*3501) which ΔGbind is even smaller with reference to that of the influenza NP418-HLA B*3501 complex (−8.3 kcal/mol) (Adhikari et al., 2018). Formation of peptide-HLA complex is more favourable as indicated by very small dissociation constants (Kd). All epitopes have good to high binding affinities (Kd < 5.0E-07M) to their HLA binders (Koyanagi et al., 2010; Paul et al., 2013). Figure 5 shows the RMSD plot per residue of each peptide-HLA complex. The mobility of a residue is often represented by its RMSD value during molecular dynamics simulation. The lower the RMSD value, the weaker the mobility, making interactions more stable. Furthermore, all complexes formed have RMSD values between 0 to 1.0 Ǻ, indicating positive and stable interactions (Fu et al., 2018). 

**Table 1:** Candidate CD4+ and CD8+ epitopes

T cell	Antigen	Epitope	Position	MHC
CD4+	MAGEA3	FPVIFSKASSSLQLV	147-161	HLA-DRB1*07:01,HLA-DRB1*09:01,HLA-DRB1*01:01,HLA-DRB1*04:05,HLA-DRB1*04:01,HLA-DRB1*15:01,HLA-DRB3*02:02,HLA-DRB1*13:02
	MAGEA3	FGIELMEVDPIGHLY	162-176	HLA-DRB1*03:01,HLA-DRB4*01:01,HLA-DRB1*01:01,HLA-DRB3*01:01,HLA-DQA1*05:01/DQB1*02:01
	MAGEA3	VLHHMVKISGGPHIS	286-300	HLA-DRB1*13:02,HLA-DRB1*11:01,HLA-DRB1*09:01,HLA-DRB1*07:01,HLA-DRB1*08:02
	MAGEA4	AESLFREALSNKVDE	102-116	HLA-DRB1*07:01,HLA-DRB1*01:01,HLA-DRB5*01:01,HLA-DRB1*04:05,HLA-DRB1*13:02,HLA-DRB1*04:01,HLA-DRB1*09:01
	MAGEA4	ELAHFLLRKYRAKEL	116-130	HLA-DRB1*11:01,HLA-DRB1*15:01,HLA-DRB1*07:01,HLA-DRB5*01:01,HLA-DRB1*12:01,HLA-DRB1*01:01,HLA-DPA1*02:01/DPB1*05:01
	MAGEA4	ERVIKNYKRCFPVIF	138-152	HLA-DRB1*11:01,HLA-DRB1*15:01,HLA-DRB5*01:01,HLA-DRB1*07:01,HLA-DRB3*02:02,HLA-DRB1*13:02
	NY-ESO-1	ESRLLEFYLAMPFAT	84-98	HLA-DRB1*01:01,HLA-DRB1*07:01,HLA-DPA1*01:03/DPB1*02:01,HLA-DPA1*03:01/DPB1*04:02,HLA-DPA1*01:03/DPB1*04:01,HLA-DRB5*01:01,HLA-DQA1*01:01/DQB1*05:01,HLA-DRB1*15:01,HLA-DRB1*09:01,HLA-DPA1*02:01/DPB1*01:01,HLA-DRB1*11:01,HLA-DPA1*02:01/DPB1*05:01
	NY-ESO-1	KEFTVSGNILTIRLT	124-138	HLA-DRB1*01:01,HLA-DRB3*02:02, HLA-DRB1*04:01,HLA-DRB1*07:01,HLA-DPA1*03:01/DPB1*04:02,HLA-DQA1*05:01/DQB1*03:01,HLA-DRB1*04:05,HLA-DRB1*11:01,HLA-DPA1*02:01/DPB1*01:01
	NY-ESO-1	RQLQLSISSCLQQLS	143-157	HLA-DRB1*01:01,HLA-DRB1*07:01,HLA-DRB1*13:02,HLA-DRB4*01:01,HLA-DRB1*09:01,HLA-DRB1*04:05
	KK-LC1	ALIVFWKYRRFQRNT	14-28	HLA-DRB1*15:01,HLA-DPA1*01:03/DPB1*02:01,HLA-DRB1*11:01,HLA-DRB5*01:01,HLA-DPA1*02:01/DPB1*05:01,HLA-DRB1*08:02
	KK-LC1	TALALVRPSSSGLIN	36-50	HLA-DRB1*13:02,HLA-DRB1*01:01,HLA-DRB1*09:01,HLA-DRB1*07:01,HLA-DRB1*11:01,HLA-DRB1*08:02,HLA-DRB4*01:01
	KK-LC1	RQKRILVNLSMVENK	76-90	HLA-DRB1*13:02,HLA-DRB3*02:02,HLA-DRB4*01:01,HLA-DRB1*01:01,HLA-DRB1*11:01,HLA-DRB1*03:01
CD8+	MAGEA3	LSRKVAELVHF	109-119	HLA-B*15:01,HLA-B*58:01,HLA-A*32:01,HLA-B*15:01,HLA-B*58:01
	MAGEA3	GSVVGNWQYFF	137-147	HLA-A*26:01,HLA-B*35:01,HLA-A*30:02,HLA-A*26:01,HLA-B*58:01,HLA-A*23:01
	MAGEA3	NWQYFFPVIF	142-151	HLA-A*23:01,HLA-A*24:02,HLA-B*15:01,HLA-A*23:01,HLA-A*24:02
	MAGEA3	YIFATCLGLSY	176-186	HLA-B*35:01,HLA-B*15:01,HLA-A*30:02,HLA-B*35:01,HLA-B*53:01,HLA-B*15:01,HLA-A*30:02,HLA-A*11:01
	MAGEA4	YTLVTCLGLSY	177-187	HLA-A*01:01,HLA-B*15:01,HLA-B*35:01,HLA-B*15:01,HLA-B*35:01,HLA-B*15:01
	MAGEA4	RVNARVRIAY	293-302	HLA-B*15:01,HLA-A*30:02,HLA-A*03:01,HLA-A*30:01,HLA-B*35:01
	NY-ESO-1	RLLEFYLAMPF	86-96	HLA-A*32:01,HLA-B*15:01,HLA-A*23:01,HLA-B*40:01,HLA-B*15:01,HLA-B*44:03,HLA-B*44:02

**Table 2:** Structural B Cell Epitopes in mvax Encompassing the Series of BL Epitopes

Structural epitope	Position	Protrusion score
Linear	462-540	0.852
Discontinuous	A:V1,A:A2,A:G3,A:Q4,A:A5,A:I6,A:R9,A:G440,A:A441,A:N444,A:R445,A:N447,A:S448,A:T451,A:N452,A:G454,A:N455,A:T456,A:N458,A:N459,A:S462,A:A463,A:S465,A:R466,A:I467,A:E469,A:A470,A:A471,A:A472,A:K473,A:A474,A:L475,A:I476,A:V477,A:F478,A:W479,A:K480,A:Y481,A:R482,A:R483,A:F484,A:R486,A:N487,A:T488,A:G489,A:E490,A:M491,A:S492,A:S493,A:N494,A:S495,A:T496,A:A497,A:G498,A:P499,A:G500,A:P501,A:G502,A:R503,A:Q504,A:K505,A:R506,A:I507,A:L508,A:V509,A:N510,A:L511,A:S512,A:M513,A:V514,A:E515,A:N516,A:K517,A:L518,A:V519,A:E520,A:L521,A:E522,A:H523,A:T524,A:L525,A:L526,A:S527,A:K528,A:G529,A:F530,A:R531,A:G532,A:A533,A:S534,A:P535,A:H536,A:R537,A:K538,A:S539,A:T540	0.809

**Table 3:** Binding Energy and Binding Affinity of Peptide-HLA Complexes

Complex	Peptide	Epitope	HLA	ΔGbind (kcal/mol)	Kd (M)
P1	FPVIFSKASSSLQLV	CD4+	HLA-DRB1*01:01	-11.9	3.90E-09
P2	FGIELMEVDPIGHLY		HLA-DRB1*01:01	-11.4	9.10E-09
P3	VLHHMVKISGGPHIS		HLA-DRB1*11:01	-11.2	1.30E-08
P4	AESLFREALSNKVDE		HLA-DRB1*01:01	-12.1	3.10E-09
P5	ELAHFLLRKYRAKEL		HLA-DRB1*01:01	-13.2	5.30E-10
P6	ERVIKNYKRCFPVIF		HLA-DRB1*11:01	-11.8	4.60E-09
P7	ESRLLEFYLAMPFAT		HLA-DRB1*01:01	-12.9	8.40E-10
P8	KEFTVSGNILTIRLT		HLA-DRB1*01:01	-10.9	2.10E-08
P9	RQLQLSISSCLQQLS		HLA-DRB1*01:01	-14	1.50E-10
P10	ALIVFWKYRRFQRNT		HLA-DRB1*11:01	-14.2	1.00E-10
P11	TALALVRPSSSGLIN		HLA-DRB1*01:01	-10.9	2.10E-08
P12	RQKRILVNLSMVENK		HLA-DRB1*01:01	-12.7	1.20E-09
P13	YIFATCLGLSY	CD8+	HLA-B*15:01	-11	1.60E-08
P14	NWQYFFPVIF		HLA-B*15:01	-13.6	2.50E-10
P15	GSVVGNWQYFF		HLA-B*35:01	-9.7	1.30E-07
P16	LSRKVAELVHF		HLA-B*15:01	-11.1	1.40E-08
P17	YTLVTCLGLSY		HLA-B*15:01	-10.2	6.80E-08
P18	RVNARVRIAY		HLA-B*15:01	-12	3.60E-09
P19	RLLEFYLAMPF		HLA-B*15:01	-14.2	9.40E-11

**Figure 5:**
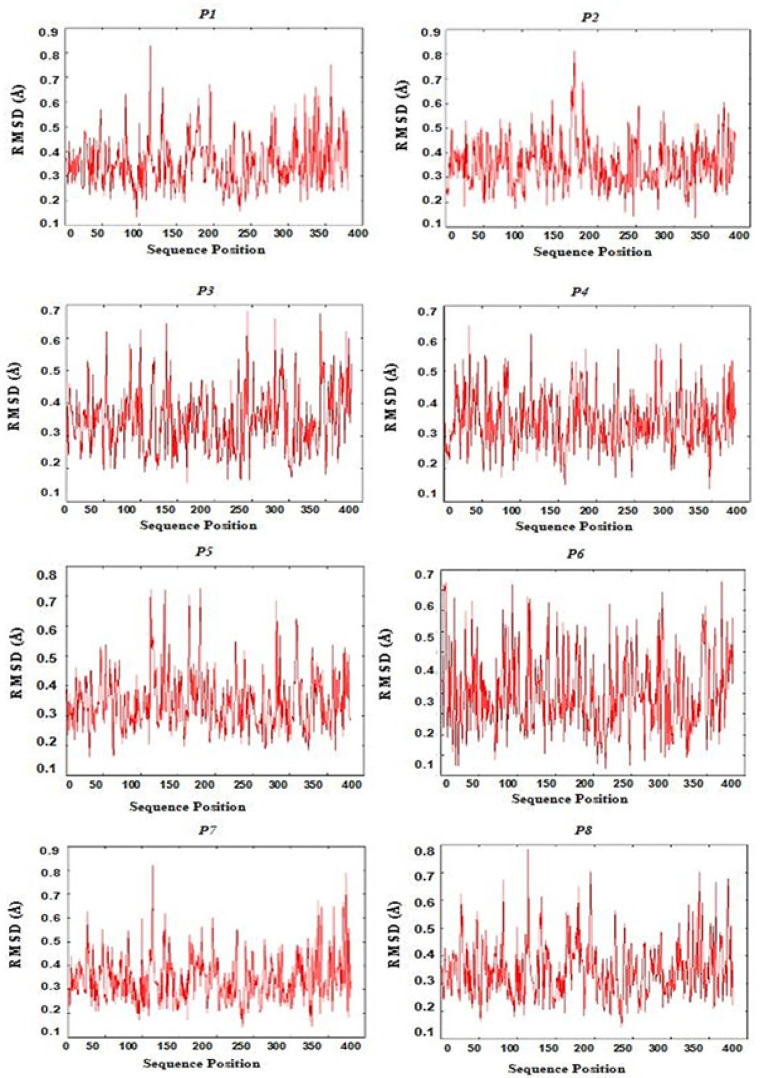
RMSD Residue Plot of Epitope-HLA Docked Structures P1-P8

**Figure 5:**
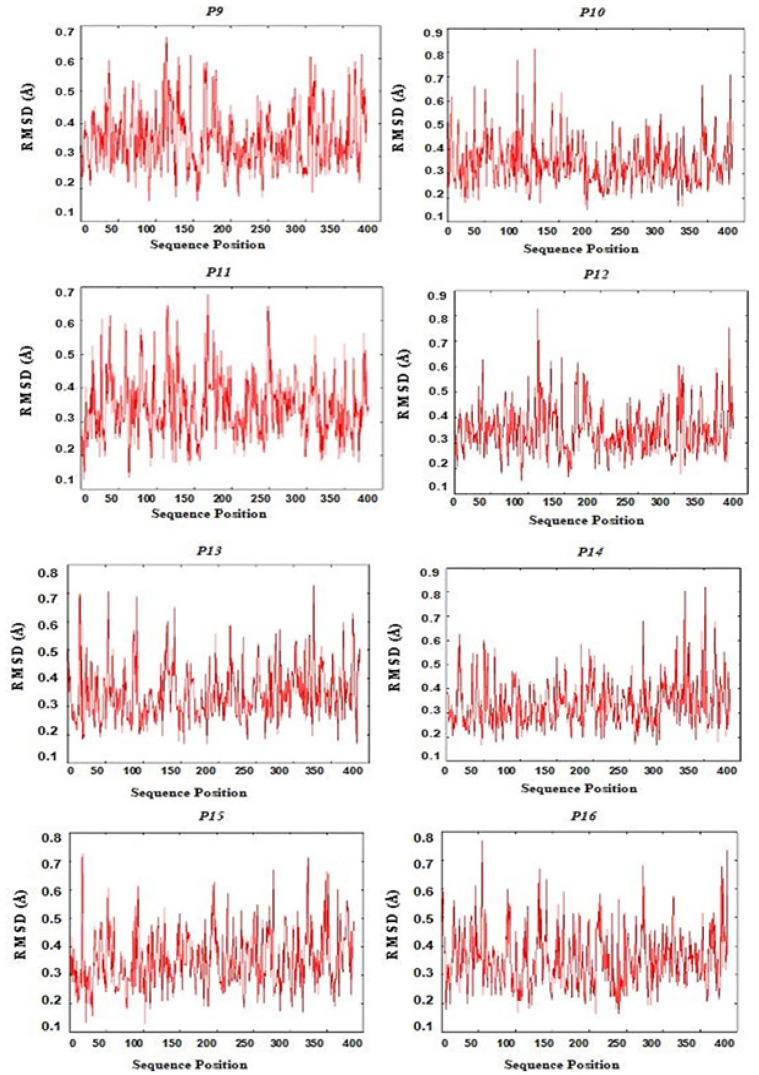
RMSD Residue Plot of Epitope-HLA Docked Structures P9-P16

**Figure 5:**
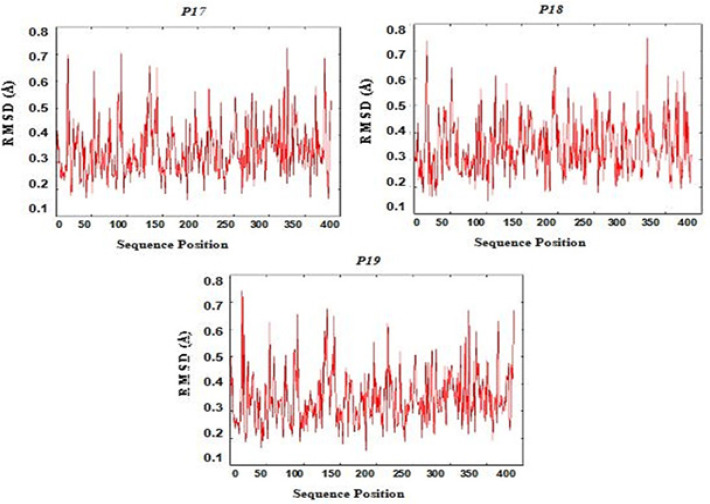
RMSD Residue Plot of Epitope-HLA Docked Structures P17-P19

## Discussion

The pathophysiology of NSCLC is made more complicated by the expression of various cancer-testis antigens (CTA) involved in its tumor-progression mechanisms. These require more extensive, yet more specific antigen-targeting approach to increase its efficacy, and to cover the majority of its cases. A clinical trial which utilized engineered T cell receptor targeting MAGEA3, lead to severe adverse events which might have been due to the cross-reaction of the peptide with MAGEA12. MAGEA12, A1, A8, and A9 were also assayed to be positively expressed in the brain in low levels (Morgan et al., 2013). A recently concluded phase III clinical trial utilized MAGEA3 as an adjuvant immunotherapeutic agent for NSCLC patients, but the disease-free survival rate did not improve (Vansteenkiste et al., 2016). Thus, this work targeted more than 1 type of antigen. The most frequently associated CTAs to NSCLC were utilized with the objective of designing a multi-epitope vaccine that can potentially induce immune responses against NSCLC expressing MAGEA3, MAGEA4, NY-ESO-1, and KK-LC1. 

Epitopes were cautiously evaluated to prevent potential cross-reaction with MAGEA12, A1, A8, and A9. Sequences with significant match to these antigens were excluded. Three BL epitopes identified from the extracellular sequence of KK-LC1 are highly antigenic, and can potentially induce humoral immune response against KK-LC1. Because this response is induced with the aid of T helper cells, CD4+ epitopes were also mapped in KK-LC1. CD4+ epitopes were identified from MAGEA3, MAGEA4, and NY-ESO-1. To be able to induce cytotoxic immune responses, CD8+ epitopes were also identified. Consensus approach was employed and at least 2 tools were utilized in the identification of all BL, CD4+, and CD8+ epitopes, increasing the accuracy of prediction. Besides good binding affinity, proteosomal cleavage, and TAP transport scores were also considered. In addition, Mvax possess large global population coverage with CD4+ (81.81%) and CD8+ (84.15%) epitopes. Some MHC binders identified in this study are not currently available in the IEDB tool, and were not included by the tool in its calculations; thus, it must be noted that the estimated %PC in this work can be larger in reality. 

The choice of adjuvant is another crucial step in vaccine design. Herein, the type of pathogen recognition receptor (PRR) activated by the adjuvant was carefully considered, as the activation of some toll-like receptors (TLRs) is associated to lung cancer progression (Chatterjee et al., 2014). On the contrary, TLR5 activation was found to have antitumor effects in NSCLC cells (Zhou et al., 2014). Flagellin is a well-studied ligand of TLR5. Due to its reported efficacy and safety, fliC was incorporated as adjuvant in Mvax. Among all 4 domains of fliC, D1 is known to be highly conserved, and has the essential interaction binding-sites to activate TLR5 (Song et al., 2017). Only D1 sequence was included to avoid possible adverse reactions. Overall, the inclusion of flagellin in Mvax may offer antitumor effects while enhancing the immunogenicity of epitopes. EAAAK linkers were used to preserve the bioactivity of fliC D1 (Arai et al., 2001). Total of 17 peptides, merged from 22 epitopes, were adjoined using AAY and GPGPG linkers known to effectively present epitopes in vivo (Jin et al., 2009). Besides efficacy, vaccines must possess safety and stability. Mvax is classified as non-allergen, and non-toxic. In addition, no sequence in the human proteome databases was found to significantly match the epitopes in Mvax, except for the 4 epitopes that match exactly with MAGEA6 and CTAG2. MAGEA6 and CTAG2 are expressed in testis, and in very low levels in placenta but are observed to be highly expressed in NSCLC and various cancer types (Wang et al., 2004; McCormack et al., 2013; Pineda et al., 2015; Tsang et al., 2020). Thus, the potential cross-reactivity of Mvax with MAGEA6 and CTAG2 may offer additional antitumor benefits. The vaccine is classified as stable (instability index <40), and can also be utilized in areas with warmer climates as it possess thermostability indicated by higher aliphatic index. In addition, the valine residue linked to the N-terminus of Mvax has notably lengthen its half-life. 

Mvax is classified as antigenic in tumor models. It has large percentage of random coils, disordered or flexible regions, and exposed sequences, more importantly within the series of B cell epitopes (Figure 2), which provide evidence for the existence of antigenic regions (Barlow et al., 1986). Antigenicity is further emphasized by the high protrusion scores of the B cell epitopes in the tertiary structure of Mvax. Moreover, the tertiary structure model used for Mvax has good and acceptable quality. The need for the refinement was validated by the significant improvements in the quality of refined tertiary model. The immunogenicity of CD4+ and CD8+ epitopes in Mvax is supported by the stability of epitope-HLA complex formed. 

In conclusion, this is the first work to use reverse vaccinology approach in designing a multi-epitope vaccine targeting MAGEA3, MAGEA4, NY-ESO-1, and KK-LC1 in NSCLC. In silico assessments showed that Mvax confers antigenicity, immunogenicity, stability, and safety. In vitro and in vivo studies are anticipated. 

## Author Contribution Statement

The author solely conducted all the requirements for the accomplishment of this work. 
